# Improvement in cardiac dysfunction with a novel circuit training method combining simultaneous aerobic-resistance exercises. A randomized trial

**DOI:** 10.1371/journal.pone.0188551

**Published:** 2018-01-29

**Authors:** Horesh Dor-Haim, Sharon Barak, Michal Horowitz, Eldad Yaakobi, Sara Katzburg, Moshe Swissa, Chaim Lotan

**Affiliations:** 1 Hadassah Hebrew University Hospital Heart Institute, Jerusalem, Israel; 2 The Edmond and Lily Safra Children's Hospital, the Chaim Sheba Medical Center, Ramat Gan, Israel; 3 Kaye Academic College of Education, Beer-Sheba, Israel; 4 Department of Physiology, Hadassah-Hebrew University Medical Center, Jerusalem, Israel; 5 Cardiac Research Center, Kaplan Medical Center, Rehovot, Israel; Texas A&M University, UNITED STATES

## Abstract

**Introduction:**

Exercise is considered a valuable nonpharmacological intervention modality in cardiac rehabilitation (CR) programs in patients with ischemic heart disease. The effect of aerobic interval exercise combined with alternating sets of resistance training (super-circuit training, SCT) on cardiac patients' with reduced left ventricular function, post-myocardial infarction (MI) has not been thoroughly investigated.

**Aim of study:**

to improve cardiac function with a novel method of combined aerobic-resistance circuit training in a randomized control trial by way of comparing the effectiveness of continuous aerobic training (CAT) to SCT on mechanical cardiac function. Secondary to compare their effect on aerobic fitness, manual strength, and quality of life in men post MI. Finally, to evaluate the safety and feasibility of SCT.

**Methods:**

29 men post-MI participants were randomly assigned to either 12-weeks of CAT (n = 15) or SCT (n = 14). Both groups, CAT and SCT exercised at 60%-70% and 75–85% of their heart rate reserve, respectively. The SCT group also engaged in intermittently combined resistance training. Primary outcome measure was echocardiography. Secondary outcome measures were aerobic fitness, strength, and quality of life (QoL). The effectiveness of the two training programs was examined via paired *t*-tests and Cohen's *d* effect size (ES).

**Results:**

Post-training, only the SCT group presented significant changes in echocardiography (a reduction in E/e' and an increase in ejection fraction, P<0.05). Similarly, only the SCT group presented significant changes in aerobic fitness (an increase in maximal metabolic equivalent, P<0.05). In addition, SCT improvement in the physical component of QoL was greater than this observed in the CAT group. In both training programs, no adverse events were observed.

**Conclusion:**

Men post-MI stand to benefit from both CAT and SCT. However, in comparison to CAT, as assessed by echocardiography, SCT may yield greater benefits to the left ventricle mechanical function as well as to the patient's aerobic fitness and physical QoL. Moreover, the SCT program was found to be feasible as well as safe.

## Introduction

Exercise is considered a valuable nonpharmacological intervention modality in cardiac rehabilitation (CR) programs designed to improve cardiorespiratory fitness and overall health status in patients with ischemic heart disease [1]. Predominantly, CR programs include continuous moderate intensity aerobic training (CAT) such as cycling and walking. CAT has been found to be effective in reducing all-cause and cardiac mortality rates [2, 3]. In contrast, patients were advocated against strenuous physical activity as it may be risky for the heart [4]. However, recently, more intensive aerobic interval programs (80%-90% of pick aerobic capacity) tested for cardiac patients and were proven safe and more effective than CAT in improving patient's cardiac outcomes and (QoL) [5].

Unlike aerobic training, until the early 1990s, resistance training was not included in the physical activity guidelines for individuals with heart conditions [3]. However, more recent guidelines do acknowledge the possible value of resistance training in CR [6]. More specifically, research shows that resistance training for individuals with cardiovascular diseases (e.g., ischemic heart disease) has numerous beneficial effects on different aspects of health (e.g., metabolic risk factors, functional capacity, psychosocial well-being) [7,8]. However, these beneficial effects may not be accountable to the central physiological mechanisms involved in left ventricle (LV) recovery post myocardial infarction (MI). Namely, during resistance training, the recruitment of large muscle groups increases vascular peripheral resistance, LV after load and local metabolic anaerobic stress. Consequently, resistance training may reduce LV wall compliance and restrict cardiac function [9]. Furthermore, animal studies and research conducted on athletes preforming moderate to high intensity exercise demonstrated enhance LV wall fibrosis and dis-synchrony [9–12]. Thus, despite the accomplishment of several studies demonstrating improved LV mechanical remodeling with aerobic training alone, combining resistance training did not demonstrate such benefits [13].

A novel type of circuit training program involves a combination of the two aforementioned training modalities, namely, resistance training set followed immediately by an aerobic exercise interval (a simultaneous aerobic-resistance training method). This type of exercise regimen commonly referred to as super-circuit training (SCT) [14]. Physiologically, SCT modality increases global metabolic and hemodynamic demand simultaneously to the anaerobic phase [14]. Thus, we presumed, it may produce favorable physiological environments during the resistance phase. Combining a sequence of resistance exercise simultaneously with aerobic exercise (i.e., SCT) is a well-established training modality in healthy athletes [15, 16]. However, the use of SCT in individuals with cardiac conditions (e.g., LV dysfunction) has not been thoroughly investigated, and may favorably induce gains in muscular endurance, strength, and cardiovascular endurance [14].

The primary aim of this study was to evaluate the effectiveness of SCT versus CAT on cardiac mechanical function (using echocardiography) in postMI patients with reduced left ventricular function (RLVF). The secondary aim was to evaluate the different effect of the two programs on non-cardiac clinical outcomes, such as aerobic functional capacity, strength, and QoL. Finally, to assess the feasibility and safety of the SCT modality. We hypothesized that in comparison to the CAT, the SCT modality will yield greater improvements in cardiac function, aerobic functional capacity, strength and QoL. Moreover, the SCT will be feasible and safe.

## Materials and methods

### Study participants

**Fig 1:**
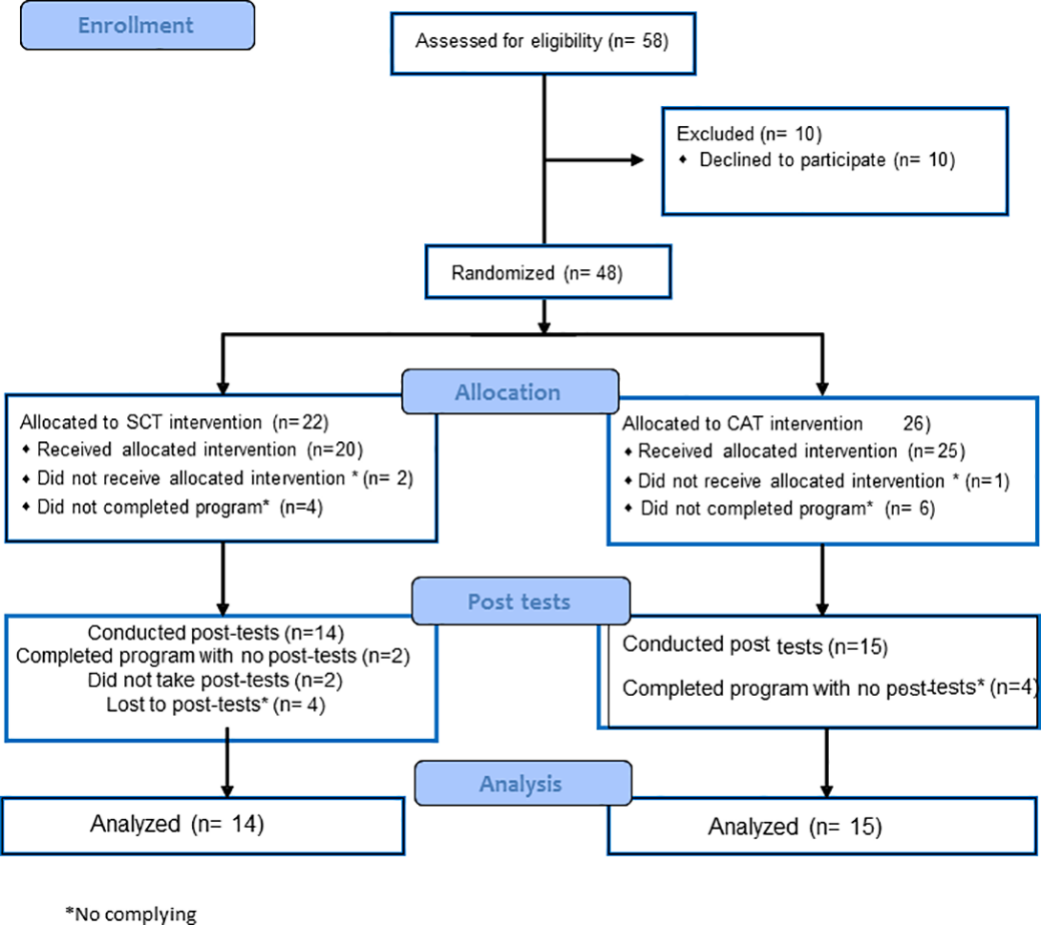
Study participant’s flow chart.

Forty-eight consecutive post-MI male patients (aged 47–69 Years old) were referred to the CR center at Hadassah Mt. Scopus 6–10 weeks post hospitalization due to acute MI (Fig 1). Patients were included in this prospective randomized controlled trial if the following criteria were met: 1) echo testing exhibited RLVF (ejection fraction < 45%): 2) patients were stable and were able to attend regularly supervised exercise program; and 4) New York Heart Association Classification I-III. Exclusion criteria were: 1) chronic atrial fibrillation; 3) severe valvular disease; 4) patients limited by angina or peripheral arterial occlusive disease; 4) cerebrovascular or musculoskeletal disease preventing exercise testing or training; 5) participants older than 80 years of age; and 6) New York Heart Association Functional Classification IV. Study participants were randomly assigned (sealed envelope method) to either twice a week SCT (n = 14) or CAT (n = 15). For information regarding the participant's clinical background, refer to Table 1. Recruiting the participants started at 7.7.13. The study ended at 29.9.14 (S1 Table). The trial ended as planned. The study was approved by the Helsinki ethics committee of Hadassah medical center (S3 File, 0440-12-HMO, ClinicalTrials.gov Identifier: NCT01912690). All participants gave written informed consent.

**Table 1:** Participant's clinical background.

group		Continuous aerobic training (n = 15)	Super-circuit training (n = 14
		n (%)	n (%)
Pharmacological treatment	Beta-blockers	10 (66.7)	8 (57.1)
	An angiotensin-converting-enzyme inhibitor	8 (81)	10 (71.4)
	Diuretics	3 (24.0)	4 (28.6)
	Statins	10 (76.7)	12 (85.7)
	Anti-coagulations	5 (33.3)	5 (35.7)
Co-morbidities	Diabetes mellitus	5 (33.3)	3 (21.4)
	Hypertension	8 (53.3)	8 (57.1)
	Obesity	4 (26.7)	3 (21.4)

### Procedures

The study was held at CR, Hadassah medical center at Mt. Scopus Jerusalem. Generation of the random allocation sequence and the enrollment of the participants was blindly done by the CR staff that were not involved in the research project. At this time, every participant received a code, that was used for reporting all the records. Assignment of participants to the different interventions was done by lottery (sealed envelope method). Study participants were randomly assigned (sealed envelope method) to either twice a week SCT or CAT. Processing and assessment of the data was done by an external employee that was not involved in the research. The care providers, eg., the cardiologist, physiologists and the coach were not blinded.

During the trial, drug therapy remained unchanged, type 2 diabetes and hypertensive patients were not regulated in their drug therapy dosage during the 12-week intervention.

### Exercise protocol

**Fig 2:**
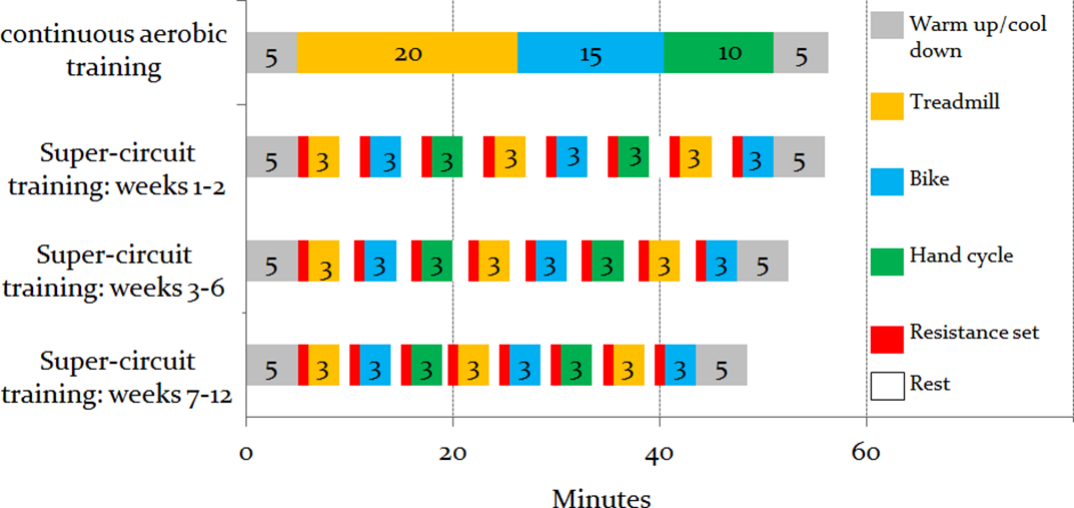
Continuous aerobic group and super-circuit group–training protocols. *Notes*: The figure illustrates the training modalities (i.e., treadmill, bike, hand cycle and resistance training) used in each training group. Each training modality is denoted in a different color. The numbers that appear inside the boxes denote the number of minutes engaged in the designated training modality; in the super-circuit group, in weeks 1–2, 3–6 and 7–12, the rest interval was of two minutes, one and a half minute and one minute, respectively.

Patients in both groups started with five minutes warm up including cycling on star trek bike and dynamic stretching. Following the warm up, participants were instructed to perform CAT or SCT (Fig 2). At the end of the training participants from both groups, conducted five minutes gradual cool down. Compared to the CAT group trained for 45 min in each session, the SCT group was trained with higher intensity and less aerobic volume. (Fig 2). Throughout the training sessions, participant's heart rate was monitored (Polar Electro, Kempele, Finland or Nihon Kohden ECG telemetry). Exercise intensity was determined using the heart rate reserved method (i.e., maximal heart rate–resting heart rate). The participant's maximal heart rate was established via a base line graded exercise tolerance test.

#### Exercise protocol–continuous aerobic training group

The CAT group participants exercised continually at 60%-70% of their heart rate reserve. In addition, the modified Borg 1-to-10 scale was used to assess the rate of perceived exertion (RPE) during and after each training session. The speed and inclination of the treadmill, or resistance and cadence of the cycle ergometer were adjusted continuously to ensure that every training session was carried out at the assigned heart rate. Blood pressure was measured before, during and at the end of each exercise session. Each session lasted 45 minutes and included 20 minutes of walking on Star Trek treadmill, 15 minutes of cycling on Star Terk bike and 10 minutes of paddling on TechnoGym hand cycle. For additional information, refer to Fig 2.

#### Exercise protocol–super-circuit training group

The SCT group preformed moderate to high intensity exercise, alternating between resistance and aerobic training (Fig 2). Each SCT set included one resistant training set, 3 minutes of aerobic interval and a resting period. This sequence was repeated eight times (Fig 2). The resistance training was composed of eight different exercises, namely, horizontal rowing, chest press, leg press, shoulder press, leg extension, lateral pull down, leg flexion and assisted squat. Each exercise consisted of one set of 15 repetitions on a Cybex machine. In the first two weeks of the program, the training intensity was light [(30% of one-repetition maximum (1RM)] and progressively increased to 50% of 1RM. Throughout the training sessions, participants were instructed to maintain appropriate lifting technique, to avoid Valsalva maneuver and to carefully change positions in order to adapt to blood pressure orthostatic changes. Each of the eight aerobic interval included three minutes of: either star trek treadmill, Star trek bike or ThechnoGym hand cycle. Aerobic intensity was designed to be 75%-85% of heart rate reserve. Similar to the CAT group, participant's blood pressure and rate of perceived exertion was monitored. Resting periods between the resistance set and the aerobic interval and between the aerobic interval and the resistance set were monitored and gradually decreased from two minutes in the first two weeks to one minute in weeks seven-to-12 (Fig 2).

### Outcome measures

Demographic and clinical background information was collected via patient's interview and from the patient's medical record. In addition, at baseline and post 12-weeks of training, participants completed a battery of tests which evaluated their cardiac function (primary outcome measures), and aerobic functional capacity, strength, and QoL (secondary outcome measures). In addition, factors related to the SCT feasibility and safety were recorded. All outcome measures were established by a blinded technician. Following is a description of the various outcome measures used in this study.

#### Primary outcome measures–cardiac function

Cardiac function was evaluated using echocardiography. Echocardiography was performed by experienced cardiologists and technician blinded to the patients’ group assignment. Participants were examined at rest in the left lateral supine position with a Vingmed Vivid 7 scanner with B-mode ultrasound at a frame rate of 50 Hz. The LV ejection fraction (EF) was calculated by Simpson’s biplane method of discs [17]. A pulsed wave Doppler was used in the apical 4-chamber view to obtain mitral inflow velocities to assess LV filling. Peak early and late diastolic transmitral flow velocities (E and A respectively) and the early diastolic wave deceleration time of the transmitral flow was measured, and the ratio of early to late transmitral flow velocities (E/A) calculated. An apical four-chamber image of the color tissue Doppler technique was acquired at a frame rate of69.8–147.7 frames/s. The peak early diastolic velocity at the mitral annuli of the interventricular septum (Se′), lateral wall (Le′) sides and IVRT were measured. Mitral inflow E velocity to tissue Doppler Le′ ratio was calculated (E/e’). The mean value of measurements was used.

#### Secondary outcome measures—Non-cardiac clinical outcomes

Aerobic fitness was assessed by using Bruce graded exercise tolerance treadmill protocol (GE Marquette CASE 8000 Exercise Testing System). From the graded exercise tolerance test the participant's pick aerobic capacity and maximal heart rate were determined. Throughout the test, blood pressure, rate of perceived exertion (Borg 1–10 scale) and 12-lead electrocardiograph were monitored. The graded exercise tolerance test was terminated if the patient presented a moderate-to-severe angina, a > 10 mmHg decrease in systolic blood pressure with increasing workload, evidence of significant arrhythmia’s (such as > 3 premature ventricular contractions in a row), evidence of poor perfusion, unusual or severe shortness of breath, equipment's mal function, or if the patient requested to stop the test. Reasons for patient-initiated termination of the test were leg fatigue, dyspnea, dizziness, or angina. In the current study, only the participant's maximal metabolic equivalent (MET) and rate pressure product (RPP) (RPP = heart rate X systolic blood pressure) are reported.

Handgrip strength was evaluated using a mechanical dynamometer. During the measurement, the palm faced inward, toward the body. The total palmar hand surface grasped the dynamometer's handle that ran parallel to the knuckles. The dynamometer faced away from the participant, such that the participant could not see and read the gauge [18]. Grip strength was used in this study as studies show that decreased grip strength is linked to a higher risk of dying from any cause, dying from heart disease, having a stroke, and a higher risk of heart attack [19]. The test was repeated three times with the dominant hand and the mean of the three tests was registered [20].

QoL was established using a general health related QoL questionnaire, the Medical Outcomes Study Short Form Health Survey (SF-12). The SF-12 contains 12 items representing eight subscales covering the domains of physical functioning, role functioning, bodily pain, general health vitality, social functioning, role-emotional and mental health. Individual subscale scores as well as two composite scores, called the physical component summary score (PCS) and mental component summary score (MCS) can be computed. In this study only the composite scores are reported, with scores ranging from 0 (the lowest level of health) to 100 (the highest level of health).

#### Super-circuit training feasibility and safety

In order to evaluate the feasibility and safety of the SCT, data regarding the program's major and minor adverse events (e.g., hospitalization, syncope, arrhythmia, muscle ache) were recorded. In addition, information regarding reasons for attrition from the program was evaluated.

### Statistical analysis

Normality assumption was evaluated using the Shapiro-Wilk test. The analysis revealed that all study variables were normally distributed. The effectiveness of the two training programs was examined via paired *t*-tests to the various study's outcome measures. In addition, differences between participants in the two training groups in the various outcome measures at both pre-and post-test were examined using independent-*t* tests. In both tests, alpha level was set to p< 0.05. As echocardiography measurement is composed of three different outcomes (E/A, E/e, EF), in order to prevent type I error, alpha was adjusted to 0.016 (0.05/3 = 0.016) using Bonferroni correction. Similarly, alpha level was adjusted to 0.025 in aerobic function (MET and RPP) and QoL (PCS and MCS) measures.

In order to quantify the degree of change in each study group with respect to the baseline values, each study group's effect size (ES) was calculated using Cohen's *d* (mean ∆/standard deviation _average from two means_) [21]. For within-participants studies, a correction for the dependence among means was conducted using the Morris and DeShon's [22] equation. In general, values smaller and equal to 0.20 are considered trivial ES, values between 0.21 and 0.50 as small ES, values 0.51–0.80 as moderate ES, and values greater than 0.80 as large ES [21].

Finally, in order to establish the SCT feasibility, the prevalence of differences in attrition rate between the SCT and the CAT programs were calculated using chi-squared test (p < 0.05). The SCT safety was evaluated via evaluation of the prevalence of minor and major adverse events in that group.

## Results

### Study participants

Forty-eight participants met the inclusion criteria for the study and were randomized allocated into CAT (n = 26) or SCT (n = 22). In the CAT group, 19 participants completed the program. However, post-test data are available only for 15 participants. In the SCT group, 16 participants completed the program. SCT post-test data are available only on 14 participants (S1 Table). For further description, refer to study participants flow-chart (Fig 1).

### Cardiac function (Echocardiography)

**Fig 3:**
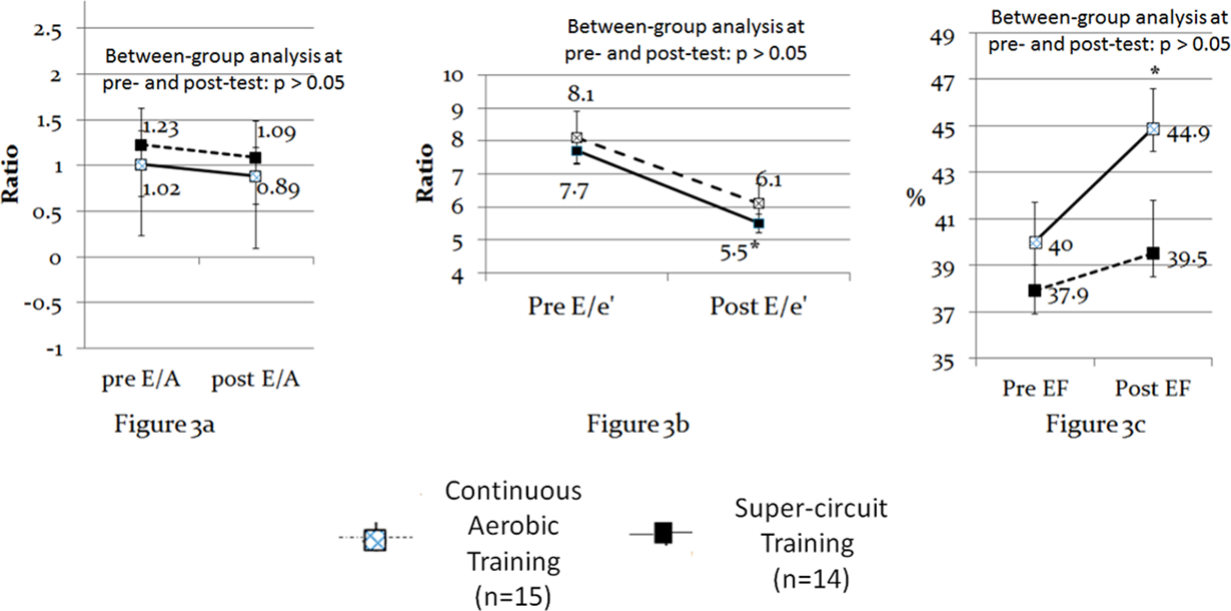
Within and between-groups differences in echocardiography. *Notes*: * significant within-group changes from pre to post-test (dependent t-test,level of significance was set at 0.05 and adjusted to 0.016, using the Bonferroni correction). No between group differences were observed (intendent t-test). ES also revealed differences between the two training modalities effectiveness. More specifically, only the SCT group presented moderate-to-large ESs (Cohen's *d* ≥ 0.51) in echocardiography measures, whereas the CAT group presented only trivial ESs in two out of the three echocardiography measures (i.e., E/A and EF) (see Table 2).

Between groups analyses revealed that at pre- and post-test there were no significant differences in echocardiography variables. Within group analysis showed that the CAT group did not present any significant changes from pre- to post-test in the echocardiography variables. In contrast, the SCT group exhibited significant changes from pre- to post-test in E/e' and EF (p < 0.016) (see Fig 3A–3C).

**Table 2:** Continuous aerobic training and super-circuit training effect sizes.

Primary and secondary outcome measures		Training group	
		Continuous aerobic training	Super-circuit training
		Cohen's *d* effect size	Cohen's *d* effect size
Echocardiography	E/A	-0.14	-0.44
	E/e'	-0.48	-0.81*
	LVEF	0.20	0.75
Aerobic capacity	Metabolic equivalent	0.36	1.08*
	Rate pressure product	-0.14	0.91*
Strength (kilograms)		1.00*	0.65
Quality of life	Physical component	0.58	1.00*
	Mental component	1.31*	0.87*

Note: Cohen's d calculation: mean ∆/standard deviation _average from two means_

* significant large difference (Cohen's d ≥ 0.80). Moderate and large differences (Cohen's d ≥ 0.51) are denoted in dark gray cells; trivial differences (Cohen's d ≤ 0.20) are denoted in light gray cells. Cohen's d is based on a single pooled standard deviation; Cohen's d was corrected for dependence between means, using Morris and DeShon's [22] equation.

### Non-cardiac clinical outcomes

Between groups analyses revealed that at pre-test there were no significant between group differences in strength and QoL (Table 3).

**Table 3:** Aerobic capacity, strength, anthropometric measurements and quality of life: within and between group differences.

			Continuous aerobic training (n = 15)			Super-circuit training (n = 14)			Between-groups analysis (independent t-test)	
			Pre-test: mean (SD)	Post-test: mean (SD)	Dependent t-test: Statistic t (p value)	Pre-test: mean (SD)	Post-test: mean (SD)	Dependent t-test: Statistic t (p value)	Pre-test: statistic t (p value)	Post-test: statistic t (p value)
Aerobic capacity	Metabolic equivalent		7.92 (2.49)	8.84 (1.72)	1.79 (0.095)	9.51 (2.17)	11.87 (1.78)	5.24 (0.002)*	1.76 (0.008)*	4.48 (0.0001)*
	Rate pressure product		18037.27 (4563.75)	17378.18 (3020.13)	-0.545 (0.597)	18902.41 (3539.09)	22143.08 (4357.47)	2.91 (0.014)*	0.100 (0.921)	3.02 (0.006)*
Strength (kilograms)			44.01 (2.42)	46.44 (2.28)	24.33 (p < 0.001)*	48.44 (9.20)	54.44 (9.58)	4.36 (0.002)*	1.39 (0.195)	2.43 (0.037)*
Quality of life		Physical component	32.51 (7.70)	36.98 (7.48)	6.40 (0.0004)*	42.35 (8.15)	50.55 (4.36)	2.92 (0.022)*	2.48 (0.026)	4.42 (0.0006)*
		Mental component	49.03 (6.71)	57.84 (4.25)	3.24 (0.011)*	38.16 (13.59)	50.03 (8.72)	3.93 (0.005)*	-2.13 (0.05)	-2.39 (0.030)

Notes: SD, standard deviation

* significant at the p < 0.05 (alpha level of aerobic capacity and quality of life was adjusted to 0.025 using the Bonferroni correction).

However, the maximal MET of the SCT group was significantly higher than the CAT group (*t* = 1.76, p = 0.008). In post-test, the SCT group performed better than the CAT group in numerous secondary outcome measures, namely, MET, RPP, and PCS (p< 0.0025) (Table 3).

Within group analysis showed that the CAT group presented significant improvements from pre- to post-test in strength, PCS, and MCS. However, the CAT group did not present any significant changes from pre to post-test in the two aerobic function parameters. Unlike the CAT group, the SCT group presented significant improvements from pre to post-test in all evaluated secondary outcome measures (Table 3). Accordingly, ES analysis revealed that in comparison to the CAT training group, the SCT group had greater impact on the secondary outcome measures. The SCT group presented moderate to large ESs in all the secondary outcome measures. In contrast, the CAT group presented moderate to large ESs only in three secondary outcome measures, namely, strength, PCS, and MCS (ES>0.58) (Table 2).

### Super-circuit training feasibility and safety

The SCT was found to be feasible. The attrition rate in CAT and SCT was similar (27% and 28%, respectively) (chi-squared = 0.114, p = 0.737). Moreover, the main cause of attrition in the SCT program was from non-program related factors such as transportation to the CR facility, difficulties in maintaining the training schedule etc., factors not related to the training program itself. The SCT was also found to be safe as throughout the 12-weeks program, no major and/or minor adverse events such as syncope, hospitalization, sever arrhythmia or disturbances in autonomic nervous system function were observed.

## Discussion

This study aimed to evaluate the effectiveness of SCT versus CAT on cardiac function, as well as on aerobic capacity, strength, and QoL in post MI patients with reduced LV function. Furthermore, to assess the safety and feasibility of SCT in those patients. Our results indicate for the first time, an improved cardiac function in patients with RLVF with the involvement of resistance training (i.e., SCT). Moreover, the SCT program was found to be feasible, safe, and showed greater effectiveness in improving patients' functional capacity.

### Cardiac function

Exercise effect on LV mechanical function is still somewhat controversial in patients' with RLVF. Numerous studies demonstrated significant increase in LV EF [23–26] and improved diastolic function with CAT. However some other studies found no improvement in LV mechanical function following CAT [27–29], even with frequent and intensive continuous training [30]. Moreover, resistance training was not associated with demonstrable benefit in LV remodeling; indeed, the favorable role of aerobic exercise was not established when it was combined with resistance training [14]. This may be because of the heightened systolic and diastolic pressure loading that occurs with resistance training [31].

In our study, echocardiographic measurements demonstrated significant improvements in ventricular mechanical function (i.e., LV EF) only in the SCT group, not in CAT group. The SCT group also significantly improved their diastolic function (i.e., a significant reduction in E/e'). The novelty in this finding is that an improvement in LV mechanical function was achieved by the practice of resistance training circuit, despite the possible LV pressure, associated with resistance training. Our results may be explained by the distinctive hemodynamic interaction of the aerobic intervals with the resistance training sets in SCT: synchronized with the peripheral vasodilatation induced by the aerobic interval, during the resistance set there is an abrupt reduction in systemic peripheral resistance and LV after load. Consequently, we assume, resistance training pressure load remodeling effect is prevented and LV compliance may increase. However, among patients limited by RLVF who were engaged in CAT, it seems likely that the increase in preload did not induce normal Frank-Starling response, suggesting that the increase in ventricular filling do not increase contractility [31]. It is also notable that compared with apparently healthy participants, vascular resistance did not decrease in response to increase in metabolic demand. This may be associated with elevated sympathetic activation [31]. Consequently, training effect in CAT may be limited, since hemodynamic responses do not engage in a concurrent increase in stroke volume. In contrast with CAT, the intermittent moderate to high intensity aerobic intervals in SCT may increase stroke volume due to intermediate decrease in peripheral resistance, and farther enhanced by skeletal muscles relaxation at the end of the resistance set.

### Non-cardiac clinical outcomes

During pre-test the functional capacity for CAT or SCT was similar except for MET, in which the SCT group presented higher values. Despite these differences during pre-test, it is not reasonable to conclude that the overall functional level of the SCT group during pre-test was better than this of the CAT group as no other significant between-group differences were observed.

Participants in the CAT group did not significantly improve in both MET and RPP. In contrast, the SCT group presented grand significant changes from pre- to post- test in both MET and RPP. These improvements were expected as the SCT group engaged in a aerobic exercise intervals. Accordingly, it has been reported that in comparison to lower intensity training, exercise performed at higher relative intensities elicit a greater increase in aerobic capacity [32]. Moreover, the SCT group also engaged in lower extremity strength exercises. It has been suggested that peripheral factors are of special importance in cardiac patients' exercise capacity [33]. Therefore, it is reasonable to assume that a combination of aerobic and lower extremity strength program in the SCT group elicited peripheral changes that in turn impacted positively the participants' aerobic capacity.

Both CAT and SCT groups significantly improved their strength performance from pre- to post-test. CAT group has improved strength although it did not perform resistant training exercises. This could be attributed to CR induced recovery of patients' physical condition as seen in the parameters measured including muscle strength. Moreover, strength performance measurements were made using the handgrip dynamometer, which is also a non-specific common index for general well-being physical state [34]. However, at post-test the SCT group strength was significantly greater than that of the CAT group. The greater mean strength in the SCT group was not surprising as SCT group engaged not only in aerobic training but also in resistance training. Nonetheless, CAT group strength ES was greater than that observed in the SCT group (Tab. 2). These results may be related to the greater strength performance variability in the SCT group as compared to the CAT group. The improvement in muscular strength is of clinical relevance as patients with cardiac diseases exhibit impaired muscular strength and skeletal muscle atrophy [35–37].

In the current study both training groups CAT and SCT, significantly improved their physical and mental QoL from pre- to post-test. Similarly, within the cardiac disease population, numerous investigators reported significant improvements in patient’s QoL following physical exercises [38, 39]. However, in post-test, PCS of the SCT group was significantly higher than that observed in the CAT group. The SF-12 items may help in understanding this between groups difference. The SF-12 includes questions pertaining to the degree in which one's health limits the ability to engage in activities such as moving a table and pushing a vacuum cleaner. Accordingly, an increase in both muscle strength and maximal MET is expected to improve the aforementioned SF-12 items. As compared to the CAT group the SCT group exhibited greater improvement in both strength and MET, such improvement may be accountable to the greater physical QoL observed in that group.

### Super-circuit training feasibility and safety

High level of physical activity is associated with low risk of mortality and cardiovascular disease [40–42]. However, it is advocated that vigorous activity may increase the risk of sudden cardiac death in susceptible persons. As in the current study no exercise related adverse events occurred in the SCT program, we concluded that the training regimen was safe. Similarly, in a study which evaluated the effects of aerobic and resistance exercise training on patients with heart conditions Maiorana et. Al, [43] reported that no significant adverse events occurred during training sessions. In a more recent study by Rognmo et al., [44], it has been reported that in a large sample of patients with coronary heart disease (n = 4,846), the risk of a cardiovascular event is low after both high and moderate-intensity exercise. Likewise, the results of our current study suggest that SCT is feasible and safe. Therefore, such program should be considered among patients with coronary heart disease.

In conclusion, this study showed that men with RLVF, post MI stand to benefit from both aerobic training alone (CAT) and SCT. However, in comparison to aerobic training alone, SCT may yield greater benefits to the LV mechanical function as well as to the patient's aerobic function, and physical QoL. Moreover, despite the moderate to high intensity used in the SCT, it was found to be feasible and safe.

## Limitations

Our current study was subjected to several limitations. Due to a limited budget, we had to limit the number of participants in the study. We therefore chose to focus on one gender group in order to maintain the homogeneity of the results. Hence, the generalizability of the results might be questioned owing to the small sample size in each study group.

In addition, central cardiac and physiological peripheral hemodynamics, were not measured for participants during the training sessions and thus should be farther examined to substantiate the mechanisms associated with SCT training affect

Last, our study was limited in time (12-weeks of CR), in which CAT did not improve cardiac function. Although number of previous studies showed that a longer period of training (6-month of CAT) could induce an improvement in left ventricular function [25, 26]. However, in our study, no significant change in LV chamber’s size was noticed. We did not detected changes in the diastolic volume whereas end systolic volume showed a tendency of improvement that was not significant. However, it is possible that in a longer period of CR exercise training, this tendency would have become significant.

## Supporting information

S1 File Consort checklist done. (DOC)

S2 File Consort chart flow. (DOC)

S3 File Helsinkidata 440 original protocol Hebrew. (DOC)

S4 File Protocol translated to English. (DOCX)

S1 Table Data plos. (XLSX)
