# Correction: Improvement in cardiac dysfunction with a novel circuit training method combining simultaneous aerobic-resistance exercises. A randomized trial

**DOI:** 10.1371/journal.pone.0204198

**Published:** 2018-09-13

**Authors:** Horesh Dor-Haim, Sharon Barak, Michal Horowitz, Eldad Yaakobi, Sara Katzburg, Moshe Swissa, Chaim Lotan

Fig 3 is incorrect. The authors have provided a corrected version here.

**Fig 3:**
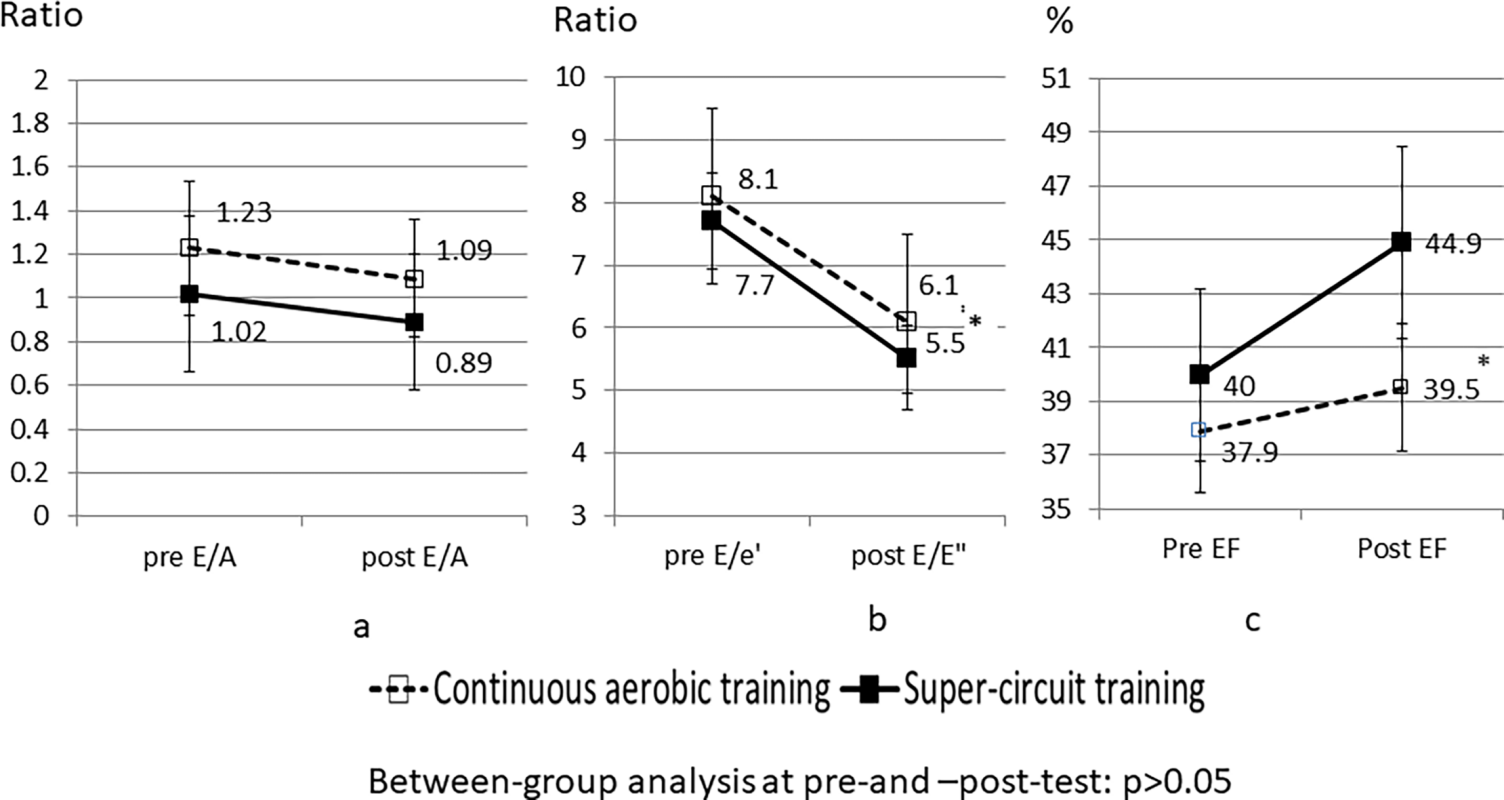
Within and between-groups differences in echocardiography. *Notes*: Data mean (SD). * significant within-group changes from pre to post-test (dependent t-test, level of significance was set at 0.05 and adjusted to 0.016, using the Bonferroni correction). No between group differences were observed (intendent t-test). ES also revealed differences between the two training modalities effectiveness. More specifically, only the SCT group presented moderate-to-large ESs (Cohen's d ≥ 0.51) in echocardiography measures, whereas the CAT group presented only trivial ESs in two out of the three echocardiography measures (i.e., E/A and EF) (see Table 2).
