# Supplementary material for: Improvement in cardiac dysfunction with a novel circuit training method combining simultaneous aerobic-resistance exercises. A randomized trial
Source: PLoS One. 2018 Jan 29;13(1):e0188551. doi: 10.1371/journal.pone.0188551 (PMC5788332; doi:10.1371/journal.pone.0188551)
Supplement: S2 File — (DOC) [file pone.0188551.s002.doc]

**Allocation**

**Analysis**

**Post tests**

**Enrollment**

Assessed for eligibility (n= 58)

Excluded (n= 10)

♦  Declined to participate (n= 10)

Analysed (n=14)

Conducted post-tests (n=14)

Completed program with no post-tests (n=2)

Did not take post=tests (n=2)

Lost to post-tests (give reasons)* (n= 4)

Allocated to SCT intervention (n=22)

♦ Received allocated intervention (n=20)

♦ Did not receive allocated intervention* (n= 2)

♦ Did not completed program* (n=4)

Conducted post-tests (n=15)

Completed program with no post-tests (n=4)

Allocated to CAT intervention (n=26)

♦ Received allocated intervention (n=25)

♦ Did not receive allocated intervention* (n=1)

♦  Did not completed program* (n= 6)

Analysed (n=14)

Randomized (n= 48)

*No complying
