# Supplementary material for: Improvement in cardiac dysfunction with a novel circuit training method combining simultaneous aerobic-resistance exercises. A randomized trial
Source: PLoS One. 2018 Jan 29;13(1):e0188551. doi: 10.1371/journal.pone.0188551 (PMC5788332; doi:10.1371/journal.pone.0188551)
Supplement: S3 File — (DOC) [file pone.0188551.s003.doc]

מספר הבקשה בוועדת הלסינקי **:**

**0440-12-HMO**

אני החתום מטה:

| שם פרטי ומשפחה: |  |
| --- | --- |
| מספר תעודת זהות: |  |
| כתובת: | מיקוד: |

1. מצהיר/ה בזה כי אני מסכים/ה להשתתף במחקר רפואי[[1]](#footnote-2), כמפורט במסמך זה.
2. מצהיר/ה בזה, כי אני משתתף/לא משתתף[[2]](#footnote-3) בזמן חתימת מסמך זה, בניסוי רפואי אחר במשך כל תקופת מחקר זה.
3. מצהיר/ה בזה כי הוסבר לי על-ידי:

| שם החוקר/חוקר המשנה המסביר: |
| --- |

כי החוקר הראשי (שם הרופא): פרופ חיים לוטןקיבל ממנהל המוסד הרפואי, בו ייערך הניסוי, אישור לביצוע המחקר הרפואי בבני-אדם, כמשמעותו בתקנות בריאות העם (ניסויים רפואיים בבני-אדם) תשמ"א-1980 (להלן הניסוי הרפואי).

כי לחוקר הראשי או לחוקרי המשנה או למנהל המחלקה **יש זיקה** ליוזם המחקר[[3]](#footnote-4).

אם יש – פרט: מדובר בחולי שיקום שמתקמים במכון השיקום בהדסה הר הצופים תחת ניהול של חוקר משנה חורש דור חיים

כי המחקר הרפואי נערך בנושא:

השפעה של פעילות גופנית על יציבות החשמלית של חולים לאחר אוטם

כי אני חופשי/ה לבחור שלא להשתתף במחקר הרפואי, וכי אני חופשי/ה להפסיק בכל עת את השתתפותי במחקר, כל זאת מבלי לפגוע בזכותי לקבל את הטיפול המקובל.

כי במקרה של מילוי שאלון – אני רשאי/ת שלא לענות על כל השאלות שבשאלון או על חלק מהן.

כי מובטח לי שזהותי האישית תשמר סודית על-ידי כל העוסקים והמעורבים במחקר ולא תפורסם בכל פרסום, כולל בפרסומים מדעיים.

כי המוסד הרפואי פעל להסדרת כיסוי ביטוחי הולם של החוקרים, הרופאים והצוות הרפואי העוסקים בניסוי הקליני מפני תביעות שיוגשו ע"י משתתפים בניסוי הקליני ו/או תביעות צד ג' הקשורות עם הניסוי הקליני בין בתקופת ביצוע הניסוי ובין לאחריו. אין באמור כדי לפגוע בזכויותיי על פי כל דין.

כי מובטחת לי נכונות לענות לשאלות שיועלו על-ידי וכן האפשרות להיוועץ בגורם נוסף (לדוגמא רופא-משפחה, בני משפחה וכו'), באשר לקבלת החלטה להשתתף בניסוי הרפואי ו/או להמשיך בו.

כי בכל בעיה הקשורה לניסוי הרפואי אוכל לפנות ל- פרופ חיים לוטן מספר טלפון/משיבון: 02-6776564 050-7874800, בכל שעות היממה.

1. הנני מצהיר/ה כי נמסר/ה לי מידע מפורט על המחקר הרפואי, על פי הנושאים המפורטים להלן:
   1. **מטרות המחקר;**

1 לבדוק כיצד מושפעת היציבות החשמלית של הלב כתוצאה מאימון גופני אירובי מבוקר במהלך תהליך של שיקום לב, לחולים לאחר אוטם?

2. לבדוק מה השפעה של אימון התנגדות משולב באירובי (אימון מחזורי), על שונות קצב הלב ועל היציבות החשמלית שלו?

3. לאפיין שינויים בכיווץ הלב, באזוריו השונים, כתוצאה מתהליך האימון לאחר האוטם, ולבחון את הקשר לתופעות של הפרעות קצב..

4. להבין כיצד משפיעות שיטות האימון השונות על היציבות החשמלית ועל שונות קצב הלב .

- 1. **התקופה הצפויה למשך ההשתתפות במחקר והמספר בקירוב של המשתתפים במחקר;**

12 שבועות של אימון ,הגיוס ומספר המשתתפים אשר ישתתפו במחקר ימשכו ככל שידרש במהלך המחקר.

- 1. **תיאור ההליכים השונים במשך תקופת המחקר, תוך הבחנה ברורה בין ההליכים המחקריים לבין ההליכים** המקובלים ברפואה;

1. המקובלים ברפואה;

קבוצות הניסוי יבצעו אימון מבוקר, לתקופה של 12 שבועות, פעמיים בשבוע במסגרת השיקום ובנוסף המלצה לבצע 30 דק' הליכה בדרגת מאמץ קל-בינוני בכל שאר ימות השבוע. האימון מתחיל תמיד ב- 5 דק' חימום קל על אופניים או הליכון ולאחריו 1-2 דק' של תנועתיות וחימום מפרקי, לאחריו 45 דק' תוכנית ההתערבות, אירובית או מחזורית ובסיום הפעילות 5 דק' של מתיחות.

קבוצת אימון הרצף: תתאמן במשך 45 דק' בדופק העומד על 60%-70% מרזרבת הדופק שיוגדר בהתאם לבדיקת המאמץ. האימון יתבצע על מכשור הבא: הליכון 20 דק', אופניים 15דק' ואופני יד 10 דק'. קבוצת האימון המחזורי: הקבוצה תבצע לסרוגין תרגילי התנגדות ותרגול על מכשור אירובי. הפעילות האירובית תכלול מקטעים עצימים קצרים של 2-3 דק': הליכון, אופניים, אופני יד, טרמפולינה, חתירה ומדרגה. המאמנים יכוונו את המכשור לעליית דופק בטווח של- 75%-85%, מרזרבת הדופק שלהם ולאחר התאוששות של 30-60 שנ', יבצעו סט של 15 חזרות, שיכלול תרגיל כוח. התרגילים יורכבו מ- 9 תחנות שונות לקבוצות שרירים שונות, לפי הסדר הבא: 1.פולי עליון 2.דחיקת רגליים 3.דחיקת חזה 4. פשיטת ברך 5.לחיצת כתפיים 6.משיכה הוריזונטלית (חתירה) 7. סקווט על כיסא 8. הנפת זרועות לצדדים 9. כפיפת ירך. רמת ההתנגדות תוגדר לכל מכשיר לפי 50% מרמת הכוח המרבית (RM1). המשתתפים יקבלו הנחיות כיצד לבצע, תנועה ונשימה נכונה, כדי לא להעלות את לחץ הדם ולהמנע מהתנגדות איזומטרית ומאפקט ולסלווה.

לפני תחילת תוכנית השיקום ובסיום השיקום יעברו כל הקבוצות, כולל הביקורת את הבדיקות הבאות: 1. איסוף נתונים אישיים 2. ECG 12 ערוצים במנוחה 3. בדיקות הולטר ECG 4. בדיקות אקוקרדיוגרפיות 5. הערכת צריכת חמצן מרבית ובדיקה קרדיופולמונרית 6. שאלון איכות חיים ובריאותSF-36 ושאלון ממוקד להערכה של הפרעות קצב ASTA145.

- 1. **היתרונות הצפויים למשתתף או לאחרים, כתוצאה מהמחקר;**

השתתפות בתכנית שיקום תסייע בהחלמה ובהקטנת גורמי סיכון , את המידע שיתקבל בניסוי לא ניתן להשיג דרך קופות חולים . ההשלכות ממידע זה יסייעו בבניית תוכנית טיפול\אימון אינדיוודואלית, המותאמת לצרכיו השונים של החולה

2. הסיכונים הידועים ו/או אי-הנוחות שניתן לחזותם למשתתף במחקר;

מידע כזה הוא בחלקו בגדר ניסוי , ולכן אינו יכול עדיין להוביל לאיבחון או לטיפול טוב יותר במצב רפואי שיתגלה , אם יתגלה . הפעילות הגופנית, יכולה לגרום לכאבי שרירים ותחושה של קוצר נשימה.

במקרים רבים המידע המתקבל הוא הסתברותי, כלומר תוצאות הבדיקה יראו שיש לך סיכוי רב יותר מאשר לאדם הממוצע (לרוב האנשים ) לחלות במחלה מסוימת , אבל אי אפשר לדעת זאת בוודאות. בנוסף לתוצאות בדיקה זו תלוי הדבר בגורמים נוספים רבים כמו סגנון החיים שלך (תזונה פעילות גופנית ) והשפעת הסביבה .

- 1. **הסיכונים הידועים ו/או אי-הנוחות שניתן לחזותם למשתתף במחקר;**

בעקבות האימונים ייתכנו קצת כאבי שרירים ועייפות .

בדיקת מאמץ קרדיופולמונריות,הבדיקה תתבצע על אופניים ארגומטריות ,כרוך בשינויים החלים בתפקוד הגוף כגון: עלייה בדופק, עלייה בלחץ הדם ועייפות שרירים, הצמדת הולטר שגורמת לעיתים לאי נוחות עקב המדבקות והאלקטרודות הצמודות לגוף במשך 24 שעות

- 1. **מידע רלוונטי אחר:**

אין

1. הנני מצהיר/ה בזה, כי את הסכמתי הנ"ל נתתי מרצוני החופשי וכי הבנתי את כל האמור לעיל. כמו-כן, קיבלתי עותק של טופס הסכמה מדעת זה, נושא תאריך וחתום כדין.
2. עם חתימתי על טופס הסכמה זה, הנני מתיר ליוזם המחקר הרפואי, לוועדת הלסינקי המוסדית, לגוף המבקר במוסד הרפואי ולמשרד הבריאות גישה ישירה לתיקי הרפואי, לשם אימות שיטות המחקר הרפואי והנתונים הקליניים. גישה זו למידע הרפואי שלי תבוצע תוך שמירת סודיות, בהתאם לחוקים ולנהלים של שמירת סודיות.

| שם המשתתף/ת במחקר הרפואי | חתימת המשתתף/ת במחקר | תאריך |
| --- | --- | --- |
|  |  |  |

במקרה הצורך[[4]](#footnote-5)

| שם העד הבלתי תלוי | מספר תעודת זהות | חתימת העד | תאריך |
| --- | --- | --- | --- |
|  |  |  |  |

הצהרת החוקר/חוקר המשנה:

ההסכמה הנ"ל נתקבלה על-ידי, וזאת לאחר שהסברתי למשתתף/ת בניסוי הרפואי כל האמור לעיל וכן וידאתי שכל הסבריי הובנו על-ידו/ידה.

| שם החוקר/חוקר המשנה המסביר | חתימתו | תאריך |
| --- | --- | --- |
|  |  |  |

1. מחקר רפואי, כולל: לקיחת דמים, שאלונים, מחקר אפידמיולוגי, מחקר בדגימות רקמה וכו', פרט למחקר גנטי. [↑](#footnote-ref-2)
2. מחק את המיותר [↑](#footnote-ref-3)
3. אם החוקר הראשי הוא גם יוזם המחקר, יש לציין זאת במפורש. [↑](#footnote-ref-4)
4. במקרה שהמשתתף בניסוי, או נציגו החוקי, אינו מסוגל לקרוא את טופס ההסכמה מדעת, עד בלתי תלוי חייב להיות נוכח במשך ההסבר על מהות הניסוי הרפואי. לאחר שהמשתתף או נציגו החוקי הביע את הסכמתו בעל-פה להשתתפות בניסוי, העד יחתום על טופס ההסכמה, תוך ציון תאריך החתימה. [↑](#footnote-ref-5)
