# Supplementary material for: Improvement in cardiac dysfunction with a novel circuit training method combining simultaneous aerobic-resistance exercises. A randomized trial
Source: PLoS One. 2018 Jan 29;13(1):e0188551. doi: 10.1371/journal.pone.0188551 (PMC5788332; doi:10.1371/journal.pone.0188551)
Supplement: S4 File — (DOCX) [file pone.0188551.s004.docx]

**Study protocol version 4 Date: 04/06/13**

This study will investigate the adjustment of the heart’s electrical system and its resistance to sudden cardiac death. It will be studied through the construction of a model for human clinical studies.

**The study population**

The study will include male patients with heart disease with systolic dysfunction of the myocardium: 1. with slight to moderate damage to the left ventricle (EF> 45). 2. patients with heart failure with functional levels II-III, with poor systolic function (moderate to severe) of left ventricular (EF <45).

Patients will be referred to our CR from hospital cardiac units and heart failure medical centers from around the Jerusalem area, as part of the cardiac rehabilitation included in the governmental health basket. Patients will undergo an initial interview and sign a consent form to participate in clinical research. Patients will not be included in this trial if the following criteria were not met: 1) participants older than 80 years of age; 2) patients limited by angina or peripheral arterial occlusive disease; 3) chronic atrial fibrillation 4) severe valvular disease; 5) cerebrovascular or musculoskeletal disease preventing exercise testing or training;

Experimental groups will randomly be distributed into two groups performing two different training methods: 1. Continuous Aerobic Training (CAT) performing continuous aerobic training protocol 2. Super Circuit Training (SCT) group that will combine resistance training with cardio aerobic exercises.

The sample size will be determined later, after a preliminary experiment with 10 patients (Pilot), during which they will perform continuous aerobic exercise and be tested for their sensitivity to electro-physiological and cardiac functional indices.

**Training protocol**

Experimental groups will perform a controlled twice a week training for a period of 12 weeks, as part of the cardio rehabilitation program and will also be recommended to perform light to moderate intense walking activity of 30 min a day for the rest of the week. The training will always start with 5 min warming up on easy bike or treadmill, followed by 1-2 minutes of warming up their motility and joints, followed by 45 min of the intervention program, or cyclic aerobic activity and at the end of 5 minutes of stretching.

**Exercise protocol – continues aerobic training group (CAT)**

In the CAT group participants will exercise continually at 60%-70% of their heart rate reserve. In addition, the modified Borg 1-to-10 scale will be used to assess the rate of perceived exertion during and after each training session. The speed and inclination of the treadmill, or resistance and cadence of the cycle ergometer will be adjusted continuously to ensure that every training session is carried out at the assigned heart rate. Blood pressure will be measured before, during and at the end of each exercise session. Each session last 45 minutes and includes: 20 minutes of walking on Star Trek treadmill, 15 minutes of cycling on Star Terk bike and 10 minutes of paddling on TechnoGym hand cycle.

**Exercise protocol – super-circuit training group (SCT)**

The SCT group will perform moderate to high intensity exercise, alternating between resistance and aerobic training. The resistance training is composed of eight different exercises, namely, horizontal rowing, chest press, leg press, shoulder press, leg extension, lateral pull down, leg flexion and assisted squat. Each exercise consists of one set of 15 repetitions on a Cybex machine. In the first two weeks of the program, the training intensity will be light [(30% of one-repetition maximum (1RM)] and progressively will increase to 50% of 1RM. Each aerobic interval includs three minutes of either: Star trek treadmill, Star trek bike or ThechnoGym hand cycle. The aerobic intensity is designed to be 75%-85% of heart rate reserve. Similar to the CAT group, participant's blood pressure and rate of perceived exertion is monitored. Resting periods between the resistance set and the aerobic interval and between the aerobic interval and the resistance set will be monitored and gradually be decreased from two minutes in the first two weeks to one minute in weeks seven-to-12. Throughout the training sessions participants will be instructed to maintain appropriate lifting technique, to avoid Valsalva maneuver and to carefully change positions in order to adapt to blood pressure orthostatic changes.

Before the start and at the end of the rehabilitation program the participants will have to go through: 1. anthropometric measures (Data collection) 2. ECG at rest (12 channels). 3. holter ECG test. 4. Echocardiography 5. ergometric stress test and evaluation of maximum oxygen consumption plus cardiopulmonary test. 6. Questionnaires: quality of life and health SF-36 questionnaire, and targeted evaluation of arrhythmia ASTA145.

**Heart rate variability HRV and autonomic balance sheet**

 The test will be conducted using a Holter ECG continuously for 24 hours. Heart rate variability indices will be calculated according to the time: RMSSD SDNN and average heart rate. Also, spectral measures will be calculated: HF, LF and HF / LF ratio indicating the intensity of the sympathetic function with respect to a pre-sympathetic.

Non-invasive measurements that predict electrical stability of the heart at in rest and activity

 Holter and stress tests will be used to quantify the incidence of arrhythmic events and other parameters, indicating the lack of homogeneity in electrophysiology. We shall examine the frequency of episodes of ventricular tachycardia (NSVT / SVT), elapsed events of atrial or flatter fibrillation (AFib / Flatter). Calculations of indices of various wave QT, the standard deviation of all intervals (QTSD). Morphological variability in the amplitude and T wavelength T (T-wave alternants), will allow us to predicts defects in the heart attributed to the arrhythmic tendency. Measuring 12 channels of ECG at rest (50mm / s) will enable the calculation (Bazett Fomula) of its dispersion of QT interval (QTd and QTc).

**Echocardiography tests**

 Will be used for measuring changes in heart function and heterogeneity in contraction indices of the heart tissue. The tests will be carried out with Vivid 7 (GE, Horten, Norway), deciphering by EchoPAC GE software. LVEF is calculated using the Simpson formula. We shall measure the morphometric indicators of heart: ventricular septal thickness, heart dimensions during diastolic and systolic times. Movement tissue (myocardial strain), will be measured with the Echo Speckle Tracking. The change in the longitudinal dimension contraction of the heart (longitudinal strain), will be received by shortening of the average systolic of 16 different segments according to the Vartdal model. Index of movement of tissue heterogeneity, will be calculated as the standard deviation of maximal shortening length of the various segments. Tissue scarred areas that will not be shorten will be discarded.

**Cardiopulmonary Stress test**

 The test will be carried out on a bicycle Ergometer, stress ranked protocol (20 W / min), using system Vmax29 (Sensor Medics). We shall test the indicators for Pick maximal stressVo2, pulmonary anaerobic threshold, 12 ECG channels, Arrhythmia at stress time and maximal oxygen pulse. The test will be performed in the presence of a cardiologist.

**Ergometer Test - ECG at stress**

The test will be performed using a treadmill, gradually amended, according to the Bruce protocol (modified Bruce), when the patient is monitored to a 12-channel set ECG. The test will be controlled by a cardiologist. Changes in ECG dynamics will be examined and blood pressure will be monitored in accordance with the accepted protocol. The test will be performed in the presence of a cardiologist.

**Statistics**

The values will be calculated as averages plus the standard deviation. T-test will be conducted to assess the level of significance of the change, in functional electro physiological measures before and after the trial for each treatment. Analysis of covariance (ANCOVA) will be carried out to compare the effectiveness of each method, in relation to the other. Pearson's correlation test will be conducted to examine the interaction between variables. Statistical significance will be defined as .p <0.05 statistical analyzes will be performed using SPSS-19 software.
